# Blood glucose/lipid metabolism alterations and their association with cancer-related fatigue in breast cancer patients undergoing chemotherapy: A cross-sectional study

**DOI:** 10.5937/jomb0-59928

**Published:** 2026-02-27

**Authors:** Yaqing Liu, Zhaoyang Huang, Qingzhong Lin, Wensi Gao, Sujuan Yang, Mei Chen, Jinxia Yang

**Affiliations:** 1 Fujian Medical University, Clinical Oncology School of Fujian Medical University, Fujian Cancer Hospital, Department of Nursing, Fuzhou, Fujian, China; 2 Fujian Medical University, Clinical Oncology School of Fujian Medical University, Fujian Cancer Hospital, Department of Breast Surgery, Fuzhou, Fujian, China

**Keywords:** breast carcinoma, chemotherapy, cross-sectional survey, cancer-related fatigue, physical fitness, quality of life, karcinom dojke, hemoterapija, poprečno istraživanje, umor povezan sa rakom, fizička spremnost, kvalitet života

## Abstract

**Background:**

To cross-sectionally investigate the correlation of cancer-related fatigue (CRF) with quality of life (QoL) and physical fitness of breast carcinoma (BC) patients during chemotherapy, so as to provide reference and guidance for the future clinical development of better medical services for BC patients during chemotherapy.

**Methods:**

From February 2023 to July 2023, 400 BC patients treated at the Breast Surgery Department of the Fujian Cancer Hospital were selected as the research subjects. Physical fitness-associated indicators were investigated, including body composition, muscle function, cardiopulmonary function, and the scores of CRF Scale, QoL Questionnaire, and Exercise Self-Efficacy Scale were investigated. Further analysis was conducted on the differences in physical fitness between neoadjuvant chemotherapy (NACT) and postoperative adjuvant chemotherapy (POAC), as well as between patients with and without exercise habits.

**Results:**

Electrocardiogram showed that 244 (61.00% ) had normal heart rhythm, but 29 (7.25%) still developed abnormal cardiac function. A greater number of patients receiving NACT showed low HDL-C than those undergoing POAC (P&lt; 0 .0 5 ). In patients with exercise habits, there was a larger proportion of reduced hemoglobin (Hb) compared with those without exercise habits, with higher QoL questionnaire scores (P&lt; 0.05).

**Conclusions:**

CRF during chemotherapy was evident in BC patients. Among them, patients undergoing NACT show lower HDL-C levels, while those with exercise habits have lower Hb and higher QoL scores.

## Abstract (sr)

**Uvod:**

Cilj je bio poprečno ispitivanje korelacije umora povezanog sa rakom (CRF) sa kvalitetom života (QoL) i fizičkom spremnošću pacijenata sa karcinomom dojke (BC) tokom hemoterapije, kako bi se pružile reference i smernice za budući klinički razvoj boljih medicinskih usluga za pacijente sa BC tokom hemoterapije.

**Metode:**

Od februara 2023. do jula 2023. godine, 400 pacijenata sa rakom dojke lečenih na Odeljenju za hirurgiju dojke Onkološke bolnice u Fuđijanu odabrano je kao subjekti istraživanja. Ispitivani su indikatori povezani sa fizičkom spremnošću, uključujući sastav tela, funkciju mišića, kardiopulmonalnu funkciju, kao i rezultate na skali CRF, upitniku QoL i skali samoefikasnosti vežbanja. Dalja analiza je sprovedena o razlikama u fizičkoj spremnosti između neoadjuvantne hemoterapije (NACT) i postopera-tivne adjuvantne hemoterapije (POAC), kao i između pacijenata sa i bez navika vežbanja.

**Rezultati:**

Elektrokardiogram je pokazao da je 244 (61,00% ) imalo normalan srčani ritam, ali je 29 (7,25%) ipak razvilo abnormalnu srčanu funkciju. Veći broj pacijenata koji su primali NACT pokazao je nizak HDL-C nego oni koji su se podvrgavali POAC-u (P&lt; 0,05). Kod pacijenata sa navikama vežbanja, postojao je veći udeo smanjenog hemoglobina (Hb) u poređenju sa onima bez navika vežbanja, sa višim rezultatima upitnika o kvalitetu života (QoL) (P&lt; 0,05).

**Zaključak:**

Hronična renalna disfunkcija (HR) tokom hemoterapije bila je evidentna kod pacijenata sa rakom dojke. Među njima, pacijenti koji su podvrgnuti neoadjuvantnoj hemoterapiji (NAHT) pokazuju niže nivoe HDL-C, dok oni sa navikama vežbanja imaju niži Hb i viši kvalitet života.

## Introduction

Breast carcinoma (BC) is one of the most serious malignancies threatening women's health worldwide, with an average of more than 1.67 million new cases annually [1]. According to the survey, the risk of BC in women over the age of 20 will continue to rise and reach its peak at the age of 45-50 [2]. Despite the emerging modern treatments for BC with the development of medical technology, the mortality rate of BC has not decreased significantly, with approximately BC-associated 525,000 deaths every year [3][4]. Chemotherapy, a commonly used scheme in modern clinical treatment of BC, greatly improves the complete resection of the tumor during surgery by reducing the tumor lesion and reduces the risk of BC metastasis and deep infiltration [5].

In the course of BC, cancer-related fatigue (CRF), quality of life (QoL) and physical fitness of patients are one of the key links affecting disease progression and therapeutic efficacy. Severe CRF can affect patients' activities of daily living (ADL) and reduce their QoL and physical fitness, which is not only detrimental to the patient's physical functions, reducing treatment effectiveness and chemotherapy tolerance, but may also induce negative emotions such as psychological fatigue and anxiety, affecting patient treatment compliance and eventually leading to poor outcomes [6]. CRF, QoL, and physical fitness of BC patients are therefore hotspots in modern BC treatment research. Although previous studies have reported related factors affecting CRF, QoL and physical fitness in BC patients, the factors involved are few and the cases are limited [7], which cannot provide better clinical reference. In this study, a large sample of data from a single center were used to conduct a cross-sectional survey on CRF, QoL and physical fitness of BC patients during chemotherapy, and the differences among different populations were compared, which can provide a reliable reference for future clinical understanding of the changes in body function of BC patients during chemotherapy.

## Materials and methods

### Participants

Based on a power analysis (α=0.05, β=0.2, effect size=0.3), a sample size of 385 was estimated. Considering a 5% dropout rate, we enrolled 400 BC patients (Fujian Cancer Hospital from February 2023 to July 2023). This single-center study may introduce selection bias, and multi-center validation is warranted. All patients received chemotherapy at our hospital. The Ethics Committee of Fujian Cancer Hospital (K2023-034-01) approved the study protocol without reservations, and all participants provided written informed consent. See Table 1 for patients' general information.

**Table 1:** Patients' general information. Note: Planned exercise for ≥20min on ≥3 days per week for at least 3 months. The intensity at least meets any of the following criteria: (1) moderate intensity: heart rate reaches (220-age) x50%-70% during exercise; (2) functional training after cancer surgery under professional guidance. Meeting the above conditions was defined as having exercise habits.

Projects		n	Percentage (%)
Age		49.83±8.29	-
Educational attainment	Undergraduate and above	28	7.00
	College or equivalent	23	5.75
	High school/secondary school/vocational	62	15.50
	Junior high school	96	24.00
	Primary and below	191	47.75
Religious belief	Daoism (Chinese system of beliefs)	2	0.50
	Buddhist	155	38.75
	Catholicism	8	2.00
	Christianity	22	5.50
	Other	5	1.25
	Not have	208	52.00
Marital status	Unmarried	6	1.50
	first marriage	347	86.75
	Remarry	12	3.00
	Divorcee	15	3.75
	Bereaved of one's spouse (literary)	7	1.75
	Other	13	3.25
Type of chemotherapy	Neoadjuvant chemotherapy (NACT)	144	36.00
	Postoperative adjuvant chemotherapy	256	64.00
Exercise habit	Have	214	53.50
	None	186	46.50

### Criteria for patient enrollment and exclusion

Inclusion criteria: ① patients receiving chemotherapy for BC; ② age range: 18-65; ③ those who provided informed consent. Exclusion criteria: ① bilateral BC; ② communication disorders or history of mental illness; ③ presence of distant metastasis; (4) those with limited mobility due to serious complications.

### Research methods

### Physical fitness survey

Data were collected from the patients after admission by uniformly trained researchers. First, the patient's 1-minute pulse count and resting blood pressure were measured. Cardiac function was monitored by electrocardiograph (EDAN SE-18). The patient was then asked to hold the wrist developer with the left and right hands to measure the grip strength, respectively, with the test run in triplicate to get the best performance. 30-second sit-to-stand test: The number of times the patient completed standing-sitting-standing within 30 seconds was calculated. 6-minute walking test (6MWT): The patient's maximum walking distance (meters) in 6 minutes at the fastest speed he/she can bear was measured on a 30-meter-long walkway.

### Laboratory tests

The patient's height and weight were measured on an empty stomach the next morning, and venous blood samples were taken to examine fasting blood glucose (FPG), hemoglobin (Hb), total cholesterol (TC), triglyceride (TG) and high/low density lipoprotein cholesterol (HDL-C/LDL-C).

FPG was measured with a blood glucose meter (Roche Accu-Chek Performa) : After 8-12 hours of fasting, fingertips were disinfected with 75% alcohol (ring finger recommended), and blood was collected with a blood sampling needle after drying (blood volume 2-3 μL). To avoid pressing hard (to prevent dilution of tissue fluid), the first drop of blood was wiped off with a cotton piece, and the second drop of blood was dropped into the blood collection area of the test strip. The test strip was inserted into the blood glucose meter and started automatically. Blood aspiration test strip reaction zone (about 5 seconds) and instrument display results (3-10 seconds). Pre-analysis: the sample type was fresh peripheral whole blood, tested within 30 minutes after collection (room temperature ≤25°C); if testing is delayed, it should be stored at 4°C (≤2 hours) to avoid freezing. The low (4.0-6.0 mmol/L) and high (8.0-12.0 mmol/L) quality control solution was used once a day before testing. The results were recorded and Levey-Jennings chart was drawn. When the quality control value exceeds the mean ±2SD, the test strip batch number, instrument status (such as electrode cleanliness), ambient temperature and humidity (ideal temperature 15-30°C, humidity ≤80%) should be checked.

An automatic biochemical analyzer was used to detect Hb, TC, TG, and HDL-C/LDL-C (Beckman Coulter AU5800) : whole blood (2 mL) anticoagulated with Ethylene Diamine Tetraacetic Acid (EDTA) was used for Hb, and serum was used for other indicators. The Calibrator (RANDOX calibrator) was used to calibrate the instrument every week, input the concentration value of calibration solution, and run the calibration program. Daily detection of low (Hb: 120—140 g/L; TC: 4.0-5.0 mmol/L; TG: 1.0—1.5 mmol/L; HDL-C: 1.0—1.2 mmol/L; LDL-C: 2.0—3.0 mmol/L), high (Hb: 160—180 g/L; TC: 6.0—7.0 mmol/L; TG: 2.0—2.5 mmol/L; HDL-C: 1.5—1.8 mmol/L; LDL-C: 3.5—4.5 mmol/L). Put the samples/calibrators/quality controls into the sample rack and edit the test list according to the item order. The instrument automatically completed the sample addition, reagent addition, reaction (37°C constant temperature), absorbance detection, calculation (based on calibration curve) and output results.

### Questionnaire survey

The research team distributed the Cancer-Related Fatigue Scale [8], Exercise Self-efficacy Scale (ESES) [9] and Quality of Life Questionnaire [10] using the online survey tool Sojump. The CRF scale consists of 17 survey items (each scored 1—5 points), with higher scores indicating more severe fatigue. The ESES includes 18 survey items, each with a score of 0—100, with the total score in direct proportion to self-exercise efficacy. The Quality of Life Questionnaire assesses the patient's QoL from four areas (physiological, emotional and functional status as well as additional items), with a total score of 100; a higher score suggests a better QoL [11][12][13]. 

### Statistical analyses

This study employed SPSS22.0 for statistical analyses. Counting data, represented by [n(%)], were comparatively analyzed by chi-square tests. Measurement data, statistically described as (χ±s), were compared between groups using independent samples t tests (after Shapiro-wilk test, all data conform to normal distribution). All pairwise comparisons were adjusted using the Bonferroni correction to account for multiple testing. The significance threshold was set at P < 0.05/number of comparisons. A minimum significance threshold of *P*<0.05 was used [14].

## Results

### Physical fitness survey results

First of all, the physical fitness survey of the 400 BC patients showed a mean BMI of (23.52 ±3.93) kg/m^2^, which was slightly higher. According to the blood testing results, Hb and FPG were mostly normal, while 73 (18.25%) had increased FPG, suggesting the possibility of diabetes. In the blood lipid examination, TG, TC, and HDL-C were elevated in 153 (38.25%), 193 (48.25%), and 169 (42.25%) of patients, respectively. Electrocardiogram (ECG) indicated normal heart rhythm in 244 (61.00%) and abnormal heart function in 29 (7.25%). In terms of HR testing, 30-second sit-to-stand test and 6MWT, the results were all in the normal range, while the grip strength of the right hand was slightly higher versus the left hand, both lower than the normal value. See Table 2 for details [15][16].

**Table 2:** Physical fitness survey results.

Projects		n	Percentage
Weight		57.31±8.41	—
Height		156.73±6.53	—
BMI (kg/m^2^)		23.52±3.93	—
Hb (g/dl)<br>(Ref: 115—150)<br>[11]	Normalcy	285	71.25
	High	0	0
	Low	115	28.75
FPG (mmol/L)<br>(Ref: 3.9—6.1)<br>[12]	Normalcy	327	81.75
	High	73	18.25
	Low	0	0
TC (mmol/L)<br>(Ref: 2.5—5.7)<br>[13]	Normalcy	193	48.25
	High	1	0.25
	Low	206	51.50
HDL-C (mmol/L)<br>(Ref: 2.07—3.37)<br>[13]	Normalcy	169	42.25
	High	6	1.50
	Low	225	56.25
LDL-C (mmol/L)<br>(reference value:<br>0.9—2.0)<br>[13]	Normalcy	3	0.75
	High	21	5.25
	Low	376	94.00
TG (mmol/L)<br>(Ref: 0—1.7)<br>[13]	Normalcy	153	38.25
	High	0	0
	Low	247	61.75
ECG	Sinus rhythm	244	61.00
	Sinus rhythm<br>+some<br>abnormalities	127	31.75
	Exceptions	29	7.25
HR (Ref: 60—100 beats/min) [15]		79.63±8.16	—
6MWT (Ref: 400—700 m) [16]		497.56±83.76	—
Grip strength<br>(Ref: 25 30 g)<br>[14]	Left hand	19.03±7.21	—
	Right hand	21.51±6.80	—
30s sit-to-stand test (Ref:<br>>17 reps is healthy) [17]		19.70±4.06	—

### Questionnaire survey results

As shown by the questionnaire surveys (Table 3), the CRF, ESES, and QoL Questionnaire scores of the 400 subjects are (29.59±11.15), (48.41±14.31) and (805.82±363.55), respectively.

**Table 3:** Questionnaire survey results.

Projects	Scores
CRF	29.59±11.15
ESES	48.41±14.31
QoL	805.82±363.55

### Differences in physical fitness of patients with different chemotherapy types

Comparing the differences in physical fitness between patients receiving NACT and those undergoing POAC, we found no marked difference in weight, height, BMI, blood glucose, ECG, cardiopulmonary function and questionnaire survey results between the two groups (*P*>0.05). In the comparison of blood lipid tests, no notable inter-group differences were identified in TC, TG and LDL-C, but a greater number of patients receiving NACT exhibited low HDL-C compared with those undergoing POAC (*P*<0.05). In addition, the left- and right-hand grip strength of POAC patients was significantly different (*P*<0.05), while there was no difference in the left- and right-hand grip strength in patients undergoing NACT (*P*>0.05), as shown in Table 4.

**Table 4:** Differences in physical fitness of patients with different chemotherapy types. Note: * indicates a statistically significant difference in right hand grip strength from the same group (P<0.05).

Projects		NACT (n=144)	POAC (n=256)	* χ^2^ (t) *	* P *
Weight		59.46±6.50	56.34±9.26	0.790	0.430
Height		155.70±10.33	157.25±4.24	1.302	0.194
BMI (kg/m^2^)		24.58±2.78	23.04±4.38	1.332	0.184
Hb (g/dL)	Normalcy	100 (69.44)	185 (72.27)	0.358	0.550
	Low	44 (30.56)	71 (27.73)		
FPG (mmol/L)	Normalcy	112 (77.78)	215 (83.98)	2.380	0.123
	High	32 (22.22)	41 (16.02)		
TC (mmol/L)	High	69 (47.92)	124 (48.44)	1.783	0.410
	Low	1 (0.69)	0 (0.0)		
	Normalcy	74 (51.39)	132 (51.56)		
HDL-C (mmol/L)	High	62 (43.06)	107 (41.80)	6.178	** 0.046 **
	Low	5 (3.47)	1 (0.39)		
	Normalcy	77 (53.47)	148 (57.81)		
LDL-C (mmol/L)	High	1 (0.69)	2 (0.78)	1.304	0.521
	Low	10 (6.94)	11 (4.30)		
	Normalcy	133 (92.36)	243 (94.92)		
TG (mmol/L)	High	65 (45.14)	88 (34.38)	4.521	0.034
	Normalcy	79 (54.86)	168 (65.63)		
Electrocardiogram results	Sinus rhythm	86 (0.69)	158 (0.78)	0.738	0.691
	Sinus rhythm +<br>some abnormalities	49 (34.03)	78 (30.47)		
	Exceptions	9 (6.25)	20 (7.81)		
HR (beats/min)		81.40±8.68	78.82±8.21	1.054	0.292
6MWT (m)		447.20±84.84	520.45±76.16	1.239	0.216
Grip strength (g)	Left hand	22.01±5.10	17.81±7.68	0.853	0.394
	Right hand	22.59±5.83	21.06±7.25*	2.110	0.036
30s sit-to-stand test		19.11±3.33	19.95±4.39	0.919	0.359
CRF		29.40±11.29	29.69±11.10	0.254	0.800
ESES		47.69±15.29	48.81±13.73	0.755	0.451
QoL		805.89±368.50	805.78±361.46	0.003	0.998

### Differences in physical fitness of patients with different exercise habits

Then, after grouping patients based on their exercise habits for comparison, we also found no difference in the survey results of weight, height, BMI, blood glucose and ECG between patients with exercise habits and those without (*P*>0.05); however, more patients with exercise habits showed lower Hb than those without (*P*<0.05), and higher QoL questionnaire scores were found in patients with exercise habits (*P*<0.001). Similarly, in patients without exercise habits, there were obvious differences in their left- and right-hand grip strength (*P*<0.05), as shown in Table 5.

**Table 5:** Differences in physical fitness of patients with different exercise habits. Note: * indicates a statistically significant difference in right hand grip strength from the same group (P<0.05).

Projects		Have (n=214)	None (n=186)	* χ^2^ (t) *	* P *
Weight		57.57±7.75	56.99±9.82	0.932	0.352
Height		156.00±5.07	157.57±8.24	0.754	0.451
BMI (kg/m^2^)		23.82±3.52	23.13±4.66	0.879	0.380
Hb (g/dL)	Normalcy	142 (66.36)	143 (76.88)	5.383	** 0.020 **
	Low	72 (33.64)	43 (23.12)		
FPG (mmol/L)	Normalcy	171 (79.91)	156 (83.87)	1.048	0.306
	High	43 (20.09)	30 (16.13)		
TC (mmol/L)	High	107 (50.00)	86 (46.24)	1.644	0.440
	Low	0 (0.0)	1 (0.54)		
	Normalcy	107 (50.00)	99 (53.23)		
HDL-C (mmol/L)	High	93 (43.46)	76 (40.86)	0.289	0.865
	Low	3 (1.40)	3 (1.61)		
	Normalcy	118 (55.14)	107 (57.53)		
LDL-C (mmol/L)	High	3 (1.40)	0 (0.0)	2.632	0.268
	Low	11 (5.14)	10 (5.38)		
	Normalcy	200 (93.46)	176 (94.62)		
TG (mmol/L)	High	75 (35.05)	78 (41.94)	1.999	0.157
	Normalcy	139 (64.95)	108 (58.06)		
Electrocardiogram results	Sinus rhythm	137 (64.02)	107 (57.53)	2.675	0.263
	Sinus rhythm + some<br>abnormalities	65 (30.37)	62 (33.33)		
	Exceptions	12 (5.61)	17 (9.14)		
HR (beats/min)		78.78±5.95	80.71±10.80	0.106	0.916
6MWT (m)		516.44±76.60	473.29±92.17	0.458	0.647
Grip strength (g)	Left hand	19.01±7.40	19.05±7.24	1.264	0.207
	Right hand	20.83±7.00	22.44±6.68	1.768	0.078
30s sit-to-stand test		19.65±4.39	19.77±3.77*	0.235	0.815
CRF		28.91±10.88	30.37±11.44	1.306	0.192
ESES		49.18±13.35	47.52±15.32	1.155	0.249
QoL		889.14±346.80	709.95±359.58	5.067	<0.001

## Discussion

As the primary malignancy that threatens women's health and life safety, how to effectively improve treatment effectiveness and patient prognosis is the focus of modern clinical research [17][18]. Evidence has shown that paying attention to changes in patients' physical fitness during treatment is helpful to assist clinical development of more targeted treatment and rehabilitation strategies [19]. In this study, we conducted a cross-sectional survey on the physical fitness of BC patients during chemotherapy, and compared the differences in physical fitness of different demographic groups, which can provide a reliable reference for clinical practice.

Four hundred cases of BC undergoing chemotherapy in our hospital were included as research subjects. Data on the basic characteristics of all the research participants revealed a median age of 48 years old, with the oldest being 69 years old and the youngest being 29, suggesting the prevalence of BC in women of all ages, which accords with the current epidemiological characteristics of BC [20][21]. Of them, 144 cases received NACT, the purpose of which was to reduce the tumor lesion by chemotherapy and facilitate the complete resection of the focus during surgery; 256 cases received POAC to further eliminate cancer cells in vivo and prevent cancer recurrence and metastasis. In the physical fitness investigation, the average BMI of the research participants was calculated to be (23.52±3.93) kg/m^2^ (reference: 18.5-23.9 kg/m^2^). Although the median does not exceed this range, it is basically close to the maximum, indicating that BC patients tended to be overweight. This can also be confirmed by the elevated TG, TC and HDL-C levels of most patients in the blood lipid examination. In addition, 18.25% (73 cases) had increased FPG, which was suspected to be complicated with diabetes; 7.25% (29 cases) had abnormal cardiac function, and 31.78% (127 cases) suffered from partial abnormal cardiac function, presumably related to adverse reactions caused by chemotherapy. Besides, the HR, 30-second sit-to-stand test and 6MWT results of all the subjects were within the normal range, while the grip strength of the left and right hands was lower than the normal value. All the included subjects had a higher right-handed grip than their left, suggesting that the majority were right-handed and therefore had a higher right-handed grip. In addition, in previous studies, researchers propose that the decline in muscle endurance of tumor patients was related to CRF, with decreased body function and vitality as the main manifestations [22]. At the same time, the toxic and side effects of chemotherapy drugs are also one of the important factors affecting the physical fitness of patients [23]. As we all know, the side effects of chemotherapy can lead to alopecia, endocrine regulation disorders, poor gastrointestinal function and so on, affecting patients' mood, sleep, nutrition intake, etc. [24], which also greatly reduces their physical fitness. And as indicated by the questionnaire surveys, the scores of CRF, ESES, and QoL of the subjects were (29.59±11.15), (48.41±14.31), and (805.82±363.55), respectively, with a higher CRF score compared with previous research [25], which also confirms the obvious CRF of BC patients during chemotherapy, consistent with our views mentioned above.

Subsequently, in order to further understand the differences in physical fitness in patients with different demographic characteristics, we conducted a comparative analysis of different chemotherapy modalities and people with and without exercise habits. The results showed that in the comparison of the two chemotherapy regimes (NACT versus POAC), the patients' weight, height, BMI, blood glucose, ECG, cardiopulmonary function and questionnaire survey results were not statistically different, but more patients receiving NACT exhibited low HDL-C levels. As we all know, the HDL-C reduction is associated with hyperlipidemia [26]. Yet, no notable difference was identified in lipid function test results between patients with NACT and those with POAC. This may be due to the chance of statistical calculation, the normal physiological reduction of HDL-C in these patients, or the destruction of the normal metabolic and immune status of the human body by chemotherapy drugs-induced toxic side effects, causing abnormal changes in lipid function [27]. However, the reduction in HDL-C observed in NACT patients may relate to chemotherapy-induced dysregulation of lipoprotein lipase activity or increased CETP-mediated lipid transfer, as previously reported in breast cancer models [28]. For validation, we will include more case data as soon as possible. Furthermore, an obvious difference was determined in the left- and right-hand grip strength in patients undergoing POAC, which is speculated to be due to the influence of the surgical side on the limb strength of BC patients [29]. However, since the surgical side of patients is not counted in this study, it is not yet possible to confirm whether the difference was induced by the surgical side, which we will supplement later.

On the other hand, we observed a lower Hb in patients with exercise habits, which is hypothesized to be related to exercise-induced destruction of red blood cells and a decrease in exercise-induced Hb [30]. However, the QoL scores of patients with exercise habits increased significantly, suggesting that maintaining exercise habits may help improve patients' outcomes. Similarly, research shows that rehabilitation exercises have a positive effect on improving the QoL of BC patients [31]. CRF, sleep disturbances and decreased cardiopulmonary endurance are common side effects of cancer treatment that can last for many years even after treatment has ended, causing considerable suffering to cancer survivors [32]. People suffering from CRF have traditionally been expected to limit their activities, reduce their energy expenditure, and rely on others for activities needed in their daily lives [33]. But a growing body of new evidence shows that moderate exercise for cancer patients can bring a number of physical and mental benefits and improve their physical fitness [34]. Our survey results fully validate this viewpoint, which also suggests that exercise rehabilitation training can be combined with chemotherapy for BC patients in the future, thus providing a more reliable guarantee for the prognosis of patients. It is worth noting that, the interaction between chemotherapy type (NACT vs POAC) and exercise habits warrants further exploration. While NACT patients showed lower HDL-C, exercise habits improved QoL regardless of chemotherapy regimen, suggesting a potential additive benefit of exercise across treatment modalities. Another important concern in this study is that the paradoxical decrease in Hb may reflect hemodilution or selective reporting bias due to fluid retention during chemotherapy, despite improvements in quality of life in patients with exercise habits. Of course, it is also possible that the patient had anemia.

As a single-center cross-sectional study, our findings are subject to selection bias and residual confounding. Key limitations include: 1) lack of healthy controls; 2) absence of data on tumor stage, chemotherapy regimens (e.g., anthracycline exposure), and concomitant medications (e.g., statins); 3) reliance on self-reported exercise habits without objective quantification. Prospective, multicenter cohorts are needed to confirm these associations. 4) due to the limited research conditions, patients were not followed up for prognosis in this study, so the impact of physical fitness differences on prognosis can not be assessed for the time being. Moreover, targeted intervention measures will be developed based on our findings and clinically trialed to further confirm the effect of physical fitness enhancement on prognosis.

## Conclusion

The physical fitness of BC patients during chemotherapy is relatively ordinary, the left- and right-hand grip strength were lower than the normal level, and the CRF is obvious. Among them, HDL-C is lower in patients receiving NACT, while Hb is lower and QoL score is higher in patients with exercise habits. Based on our findings, we recommend structured aerobic exercise (e.g., 30-min brisk walking 5 days/week) combined with resistance training (2 sessions/week) to enhance QoL in BC patients undergoing chemotherapy.

## Dodatak

### Ethical approval

The Ethics Committee of Fujian Cancer Hospital (K2023-034-01) approved the study protocol without reservations.

### Consent to publish

All authors gave final approval of the version to be published.

### Competing interests

The authors report no conflict of interest.

### Availability of data and materials

The data that support the findings of this study are available from the corresponding author upon reasonable request.

### Funding

Science and Technology Program of Health Commission of Fujian Province: Medical Innovation Project (No. 2022CXA030).

### Acknowledgements

Not applicable.

### Author contributions

YQ.L. conceived and designed the study, YQ.L. and ZY.H. wrote and revised the manuscript, QZ.L. and WS.G. collected the data, SJ.Y and M.C. analyzed the data, QZ.L. visualization the data, JX.Y supervised the study, YQ.L. and ZY.H. Made equal contributions to this work as co-first authors. All authors read and approved the final submitted manuscript.

### Conflict of interest statement

All the authors declare that they have no conflict of interest in this work.
